# Exploring a holistic training program on tactical behavior and psychological components of elite soccer players throughout competition season: a pilot study

**DOI:** 10.1186/s13102-024-00811-x

**Published:** 2024-01-22

**Authors:** Juan M. Tassi, Hadi Nobari, Jesús Diaz García, Ana Rubio, Miguel Ángel López Gajardo, David Manzano, Tomás García-Calvo

**Affiliations:** 1 Faculty of Sport Sciences, University of Extremadura, 10003 Cáceres, Spain; 2 Department of Exercise Physiology, Faculty of Educational Sciences and Psychology, University of Mohaghegh Ardabili, 56199-11367 Ardabil, Iran

**Keywords:** Stress, Adversity, Sport aggressiveness, Principles of play, Tactical component, Competition

## Abstract

This study aimed to assess the effects of a training program, considering an ecological/holistic perspective, on both tactical behavior (i.e., principles of play; PP) and psychological aspects (i.e., emotional, and cognitive components) in elite soccer teams consisting of players from the U23 and U21 age groups. The participants were 46 players from the under U-23 and U-21 teams from the same club and the first division of Argentina. A quasi-experimental design was examined after five weeks of intervention through integrated training tasks, where psychological factors were used for the development of the tactical principles of the game. Two evaluations of the improvement of game principles were performed before and after the intervention. The results showed significant changes and differences in both teams concerning the PP during the post-intervention period. Specifically, the results show significant group-by-time interactions with an increase in the percentage of tactical actions and behaviors performed during the competitions in two game principles analyzed in both teams. There were significant in the group-by-time interactions for PP 3 (i.e., the first option to pass forward; *p* ≤ 0.001, F = 58.96, ηp^2^ = 0.88) and also, significant changes were in PP 4 (i.e., immediate pressure when losing the ball) through the main effect of time (*p* ≤ 0.001, F = 105.41, ηp^2^ = 0.93) and group by time interactions (*p* = 0.002, F = 20.08, ηp^2^ = 0.72). In both groups, there were significant changes in post hoc analysis (PP3: U21: *p* = 0.039 vs. U23: *p* ≤ 0.001) and (PP4: U21: *p* = 0.006 vs. U23: *p* = 0.001). It seems the strategies and constraints used, tactical components, and integrated into the psychological aspects during specific soccer training tasks can help improve the tactical behaviors of both teams in a competition associated with the PP of a soccer team.

## Introduction

Despite sports psychology’s recognition of performance, many psychological aspects and abilities have yet to be incorporated into specific training tasks in recent years [1–3]. In that sense, some studies have used strategies and constraints to improve psychological and mental aspects under stressful situations or training under pressure in a general way [4–8]. Specifically, the physical was influenced by training under exhaustion conditions. For example, the mental was influenced by the effects of tasks on the player, the environment by introducing diversions, and the task by establishing a match’s demands.

In recent decades, ecological theories have sought to develop intervention programs to improve sports’ tactical and technical aspects and the emotional and cognitive aspects associated with performance. In this, generating a positive adaptation to stress exposure and improving attentional capacities in real soccer game situations could favor the integration and development of psychological capabilities [9] linked to behaviors of team tacticians.

In this sense, ecological theories of training, as expounded by influential researchers such as Gibson [10], Renshaw et al., and Davids et al. [11, 12], highlight perception’s critical role in shaping human actions. These theories provide a foundational framework for crafting intervention programs aimed at elevating technical and tactical performance within competitive soccer. This approach extends beyond conventional coaching methodologies by acknowledging the dynamic relationship between athletes and their environment. Rather than a top-down instructional model, the ecological approach emphasizes creating training environments that simulate the unpredictable nature of actual game scenarios. It aims to develop athletes’ adaptability by considering factors like sensory information, field conditions, opponents, and teammates. Moreover, ecological training programs extend their focus beyond pure physical skill development. They incorporate psychological conditioning elements essential for comprehensive performance enhancement. These elements encompass emotional aspects like stress management, dealing with adverse situations, and handling pressure scenarios during training and competition. Additionally, cognitive aspects are addressed, including the use of external focal points and refining specific attentional mechanisms crucial for performance optimization [13]. In this context, the primary objective of intervention programs is not solely skill enhancement but also the efficient management of mental load during training sessions. By integrating emotional and cognitive elements into training interventions, coaches adopting an ecological approach aim to optimize athletes’ mental readiness and resilience during competitive engagements. This shift from conventional teaching methodologies to an ecological approach reorients coaching philosophies towards a more holistic and adaptive training model. It emphasizes creating athletes who possess not only technical proficiency but also the perceptual-cognitive skills necessary for agile decision-making and adaptability within the unpredictable dynamics of competitive sports.

Fundamentally, knowing the consequences it can have on performance [14]. These could favor the behavior and reactions expected from soccer players during different situations of stress and adversity in the competition. In that line, planning training and managing the emotional load would positively solve the different situations and scenarios of adversity in the sports context [15]. Emotional load, in this context, refers to the emotional stress experienced by players during high-pressure situations in soccer matches, and it is considered a key driver of our intervention and its outcomes. This construct generally has been used to understand how athletes or sports teams can achieve or maintain a positive adaptation despite exposure to stress or adversity [2]. In this case, it could favor the levels of activation required for a given game action (linked to defensive sport aggressiveness), which allows them to recover the ball without committing infractions, for example.

This study aims to add new knowledge to the academic literature on intervention programs based on the ecological perspective of soccer training. As seen in the research field, no known studies simultaneously allow improving tactical behavior [16–18] and capabilities related to emotional control and the development of cognitive factors during soccer competition season. In this respect, other interventions in sport psychology [19] highlighted the importance of linking technical-tactical, physical, physiological and psychological elements that allow athletes to compete with intensity and cope with pressures correctly. From that perspective, this research aims to implement an intervention program to improve tactical behaviors during competition season through strategies and constraints that associate game principles with tactical components, stress and adversity scenarios as a psychological/emotional component, and specific attention as a psychological/cognitive component. So, this may favor using sports training methodologies that integrate and develop the tactical and psychological components simultaneously and specifically.

Thus, this study will develop a pilot intervention program based on psychological aspects with different technical and tactical training tasks. Mainly, it focused on emotional variables related to stress scenarios and coping with adverse situations and cognitive variables related to specific attention. Therefore, the study’s main objective is to analyze a holistic intervention program’s effect on integrated tactical and psychological aspects of tactical performance during competition. We hypothesized that the intervention program might affect tactical behavior, like principles of the game, and psychological aspects, such as emotional and cognitive components, in elite soccer players throughout the competition season.

## Materials and methods

### Participants

**Fig. 1:**
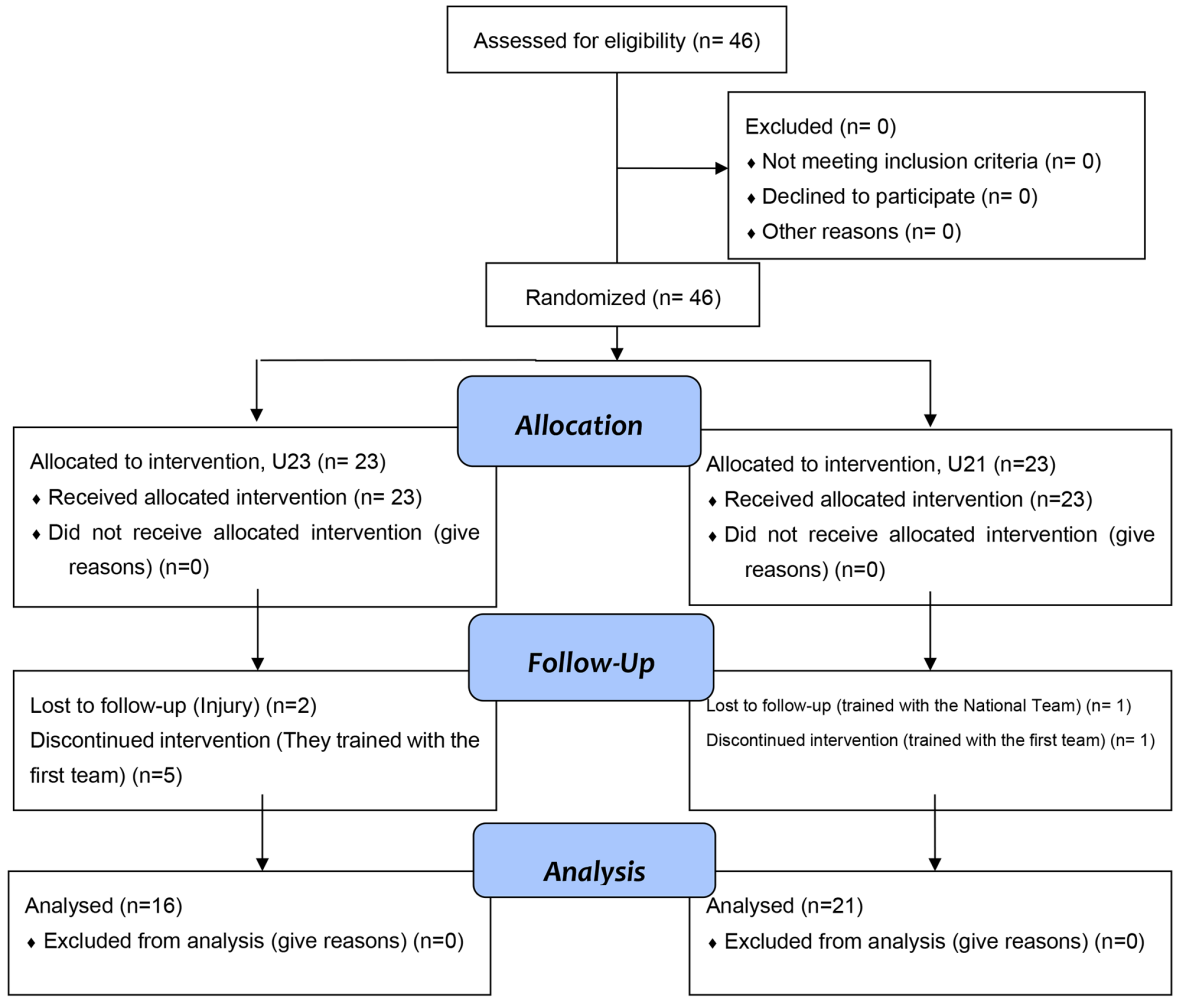
CONSORT diagram of study for each team

The study involved two grassroots soccer teams from the exact first division club in Argentina, made up of an under-23 team with 23 professional players (age: 20.61 ± 1.57and experience: 11.58 ± 2.27years) and an under-21 team with 23 professional players (age: 19.21 ± 1.03 and experience: 9.16 ± 2.59years). The selection of the participating players was carried out through purposive selection sampling. The inclusion criteria were as follows: (i) players should be ≥ 18 years, (ii) playing in the national category and with a minimum experience of 6 years playing soccer. The exclusion criteria were:(i) players who have completed less than 80% of the sessions with the team, (ii) players who have participated in training sessions with the first team, and (iii) players who have participated in training with national teams. Some players from each team were excluded due to exclusion criteria, which can be seen in Fig. 1. Consequently, the final sample comprised 16 and 21 players from the U-23 and U-21 teams.

After coordinating with the team’s staff, all the players were presented with the advantages and disadvantages of the study. Then all players signed the consent form. The study followed the Declaration of Helsinki guidelines for human studies in all phases. Before starting the study, the ethics committee of the University of Extremadura approved this study (156/2021). ClinicalTrials.gov Protocol Registration and Results System (PRS) have registered in Release Date: June 15, 2023. All data were treated according to the privacy, ethics, and protection policies of the American Psychological Association.

### Study design

This research conducted a quasi-experimental intervention design at a longitudinal level, the study used an uncontrolled two group pre-posttest design. The intervention program was based on analyzing and integrating tactical behaviors (game principles) and psychological aspects/variables associated with stress scenarios, coping with adverse situations and specific attention work. Then, different strategies to be used during specific soccer training tasks were established under a design used by the coach to mentally challenge the players [5]. There, the goal was associated with being able to challenge and stress players to develop psychological and mental aspects (e.g., attention and concentration, self-talk, arousal control) due to the manipulations included during the training tasks [20]. Fundamentally, the scenarios during the training tasks were associated with causing states of mental fatigue linked to tactical, technical performance [21], and fundamentally oriented to (i) Divide attention and maintain high levels of concentration concerning the ball, teammates, rival, playing space and change of rules during soccer training tasks; (ii) Establish objectives and physical, technical and tactical challenges during the tasks, promote confrontations in numerical inferiority, encourage stress and adversity scenarios through sanctions associated with the game (i.e. taking possession of the ball from a team), and favor actions of the game that promotes fighting and melee aggressiveness with the rival (i.e. duels).

In Table 1, it is possible to observe the explanations and relationships between tactical and psychological behaviors, seeking as an objective within the training tasks that both behaviors were united. After this, the study’s first author and the coaching staff of the different teams organized by consensus the moments of data collection and the introduction of the specific constraints 30 days before the beginning of the present research. The study employed two experienced observers who assessed each session, and both observers had several years of experience in soccer. The intervention program implemented consisted of three stages: (a) an introductory and theoretical stage, where all the information was presented: the objectives of the study, the tasks and dynamics to be developed, as well as the duration of the program; (b) the experimental stage, where the coaches received detailed training to develop the intervention program; (c) the intervention stage, where the specific program was developed by the coaches and implemented with the players of both teams. The first two phases were conducted in three 2-hour meetings [22], and the third phase lasted five weeks. There, the two teams trained under the same plan and task design five days a week, performing 25 training sessions. During this period, psychological strategies and constraints were incorporated into the technical-tactical training tasks. Specifically, before the intervention, technical-tactical behaviors and actions were analyzed during the first five games of both teams (from week 1 to 5 inclusive). During the following five weeks (weeks 6 to 10 inclusive), the strategies and constraints were used in the tasks established by the coaches, with no record of competitions during this period. Finally, the behaviors and technical-tactical actions of the following five weeks (from week 11 to 15 inclusive) were analyzed without intervention in the training tasks. The first measure, between competitions 1 and 5 inclusive, was used to determine the percentage of actions performed by both teams in the competitions without psychological conditioning during the training tasks. Subsequently, the coaches’ intervention in soccer-specific training tasks was performed between weeks 6 and 10 inclusive, using the integrated tactical and psychological constraints designed by the experts. Finally, measurements were performed during the post-intervention period to evaluate and know the effects of the intervention on the percentage of actions performed during competitions 11 to 15 inclusive (Table 1).

**Table 1:** Principle of the game and behaviors linked to psychological aspects: constraints associated with stress scenarios, coping with adverse situations and specific attention during training

Principle of the game	Description	Related psychological aspects/variables	Constraints
1- Take the ball out of the pressure zone.	1.- Take the ball out of those sectors where it is recovered.	Perceptual/attentional aspects with the ball: A.- External focus on the number of teammates and opponents in the area where the ball is located. B.- External focus of attention on the search for a free teammate to pass the ball. Context and stress situations: A.- Obligation to maintain ball possession (pressure scenario) for a given period (minimum/maximum number of passes). B.- Penalizing the non-compliance of instructions (loss of the ball, for example).	A.- Hold the ball in a zone of numerical superiority until the defense/opposition equals the number of players. A.2.- Take away the possession of the ball from the team that keeps it in a zone of numerical inferiority/equality. It is penalized with a goal against, in addition. B.- Passing to a teammate who receives the ball unmarked adds a goal in favor. B.1.- Pass to the teammate in numerical inferiority zone, causes loss of ball. C.1.- Maintain possession of the ball in a defensive playing space and with numerical superiority (own goal), until the opponent has the same number of players in this zone, there, the player will try to progress with the ball by driving or passing to the next sector to score a goal. Failure to comply with the slogan or losing the ball, will add a goal to the opposing team. C.2.- Maximum two passes between teammates, in the area where the ball is recovered. Failure to comply with the slogan, generates the loss and goal for the opposing team.
2- Mark in attack	The defenders must mark their closest rivals with the team in possession of the ball and in the opponent’s end zone (opponent’s field).	Perceptual/attentional aspects without the ball: A.- External focus of attention placed on ball location and opponent/opponent (possible receiver to score in case of losing possession). Context and stressful situations: A.- Penalizing non-compliance with instructions (removing defenders, playing in inferiority). B.- Individual responsibility to prevent the opponent from advancing and scoring in own goal. C.- Actions of individual fight/aggressiveness and melee with the rival.	A.- Failure to anticipate the forward/forward (prevent the ball from reaching the opponent) disqualifies him and eliminates him from that action. B.- Removing the defender that allows the rival striker to turn and stay in front of his goal to finish. C.- Remove the defenders that allow the attackers to receive the ball alone. C.1.- Count the number of balls received by attacking players (midfielders or strikers) as a goal. To provoke frustration in defenders. D.1.- Set up transitions of play, attack/defense and defense/attack.
3- First option to pass forward	In possession, look for unmarked teammates, in front of the line of the ball (closer to the opponent’s goal) and with the option to receive in free spaces or unmarked.	Perceptual/attentional aspects with the ball: A.- External focus of attention placed on the spaces and teammates in front of the ball line and the opponent/opponent. Context and stressful situations: A.- Playing the ball to sectors with higher risk of losing possession, due to rival Sup. Numeric Sup. Numerical. B.- Time limitations and rules to comply with the instructions. C.- Penalties for non-compliance.	A.1.- Establish a certain time to get from one sector to another. A.2.- Deliver the ball to an unmarked teammate toward the opponent’s goal. B. Each pass made towards the playing area in progression and received by a teammate adds up to a goal. B.1.- Take away possession when the pass is backwards, without pressure or opposition that forces. C.- Use a zone of numerical superiority (intermediate zone or midfield) to go out utilizing an oriented control. It adds a goal to perform this action. C.1- Do not allow in the tasks; return to the areas where the ball has already passed.
4- Immediate pressure when the ball is lost	Try to recover the ball as soon as possible.	Perceptual/attentional aspects without the ball: A.- External focus is placed on the ball, the opponent in possession, and possible receivers nearby. Context and stressful situations: A.- Maximum demand and intensity in physical actions until recovering/provoking loss of the ball. B.- Time limitations to comply with the instructions. C.- Penalties for non-compliance.	A.- Avoid the ball’s exit from the sector where the loss occurs. If so, they receive a goal against or keep defending for 1 min. B.-Recover the ball after a turnover before a certain time (6/8’’).

### Instruments and measurements

Sony Vegas Pro 13 software was used to evaluate tactical behaviour competition, allowing quantitative and qualitative analysis of the images recorded. An observation and registration form designed by the experts mentioned above was used to quantify the players’ game actions related to each match’s tactical and psychological components. For the statistical analysis of the tactical behaviors, SPSS 25 software was used.

For this objective, the observation sheet was based on four tactical principles of the game to check if the players were developing the requested behavior. Based on these principles, four-game situations were established to be evaluated, which were to be carried out during the competitions played by the team (Table 2): 1.- Taking the ball out of those sectors where it is recovered; 2.- Marking in the attack: Faced with the loss of the ball, the defenders of our team had to be marking the closest rivals when they receive the ball; 3. - First option of forwarding pass: the player who has the ball under control and with an unmarked teammate in front of him, should try to play it to that teammate as the first option; 4.- Immediate pressure and recovery after a turnover: when the ball is lost, he or the closest players should react immediately, through movements and physical actions of high intensity.

**Table 2:** Observation and recording sheet of integrated tactical and psychological behaviors during competitions

Principle of the game	Game situation	Action assessed	Realized	Not Performed	Total	% Realized
1- Take the ball out of the pressure zone.	1.- Before recovering the ball in any part of the field	1.- Take the ball out of those sectors where it is recovered. This must be done by giving a maximum of two passes between teammates within the recovery space.	1.- Yes (+)	2.- No (-)	xxxxxxxxxx	***********
2- Mark in attack	1.- With the team in possession of the ball and in the opponent’s end zone (ball in the opponent’s half), the defenders must mark their closest opponents.	1.- When the ball is lost: A.- The defenders of our team must be marking the closest rivals when they receive the ball.	1.- Yes (+)	2.- No (-)	xxxxxxxxxx	***********
3- First option to pass forward	1.- In possession, search for teammates free of opposition and marking, ahead of the line of the ball (closer to the opponent’s goal).	1.- The player who has the ball under control must play it forward as the first option, if he has an unmarked teammate.	1.- Yes (+)	2.- No (-)	xxxxxxxxxx	***********
4- Immediate pressure when the ball is lost	1.- Try to recover the ball as soon as possible.	1.- When the ball is lost, the closest player(s) must react immediately, by means of high intensity physical movements and actions (accelerations and physical contact with the opponent without committing fouls).	1.- Yes (+)	2.- No (-)	xxxxxxxxxx	***********

In all cases, the game situations were evaluated through the category of “Performed (+)” and “Not Performed (-)” according to the objective of this research. That is to say, the total of game actions to be performed in the competitions was added and the percentage of the actions performed (+) was obtained. The objective was linked to the actions and behaviors that were intended to be developed beyond the effectiveness and the result of each action. It was evaluated whether the actions were performed (for example, taking the ball out of those sectors where it is recovered), regardless of the effectiveness or result obtained (whether a teammate in another sector received the ball).

The evaluations were conducted in two-time steps in the following order: (i) the first evaluation was conducted between games 1 to 5 as pre-intervention, (ii) the second evaluation was conducted between games 11 and 15 as post-intervention. The observation sheet of both teams was used to measure the players’ behavior during the competition. A baseline measurement (before the intervention, during competitions 1 to 5 inclusive), and a post-measurement (during competitions 11 to 15), after the intervention process carried out between competitions 6 and 10 inclusive.

In Table 3, it is possible to observe how the different stages and measures used during the intervention program were proposed. The first measure, between competitions 1 and 5 inclusive, was used to determine the percentage of actions performed by both teams in the competitions without psychological constraints during the training tasks. Subsequently, the coaches’ intervention was carried out in the soccer-specific training tasks between weeks 6 and 10 inclusive, using the tactical and psychological constraints integrated and designed by the experts. Finally, measurements were taken during the post-intervention period to evaluate and know the effects of the intervention on the percentage of actions performed during competitions 11 to 15 inclusive.

**Table 3:** Measures and intervention during a holistic program implementation

Pre Intervention Week 1 to 5	Throughout Intervention Program Week 6 to 10	Post Intervention Week 11 to 15
Baseline measurement of tactical behaviors From competition 1 to 5, inclusive Only tactical constraints were used in the training tasks, i.e., no psychological or psychological constraints.	No recording or measurement of tactical behavior. Use of tactical and psychological constraints in training tasks	Post-intervention measurement of tactical behaviors From competition 11 to 15 inclusive Only tactical constraints were used in the training tasks, i.e., no psychological or psychological constraints.

### Data analysis

The SPSS 25.0 and GraphPad Prisma 9.4.1 software’s were used to handle and analyze the data. Prior to running the parametric tests that require normally distributed data, assessments of data normality were conducted. There, descriptive statistics were carried out for each team, and each game principle was represented in each competition. On all parameters, a factorial ANOVA with repeated measurements was conducted. In comparison, the between-group component considered groups (i.e., U 21 and U23), and the within-group factor considered time (i.e., pre- or post-test). It also has been calculated for the partial eta effect size (ηp^2^). If a significant time-group interaction was found, each group was subjected to a one-way repeated-measures ANOVA with a Bonferroni post hoc analysis. Then we made a comparison with Paired Samples T-Test to merge considering both groups with each other. We utilized an effect size of Hedge’s g type with a 95% confidence interval to calculate the magnitude of pairwise comparisons for the pre-and post-test. We classified it as trivial, < 0.2; small, ≥ 0.2; moderate ≥ 0.5, and large for more than 0.8. All significance levels were considered at *p* < 0.05.

## Results

There were significant main effects of time for change in principle of game number 1 (i.e., getting the ball out of the pressure zone) (*p* ≤ 0.001, F = 55.96, ηp^2^ = 0.875), however, it was no significant in group-by-time interactions (*p* = 0.965, F = 0.002, ηp^2^ ≤ 0.001). Regarding game principle number 2 (i.e., mark in attack), no significant changes were found in interaction (*p* ≤ 0.001, F = 55.96, ηp^2^ = 0.875) and time (*p* = 0.071, F = 4.34, ηp^2^ = 0.352).

**Fig. 2:**
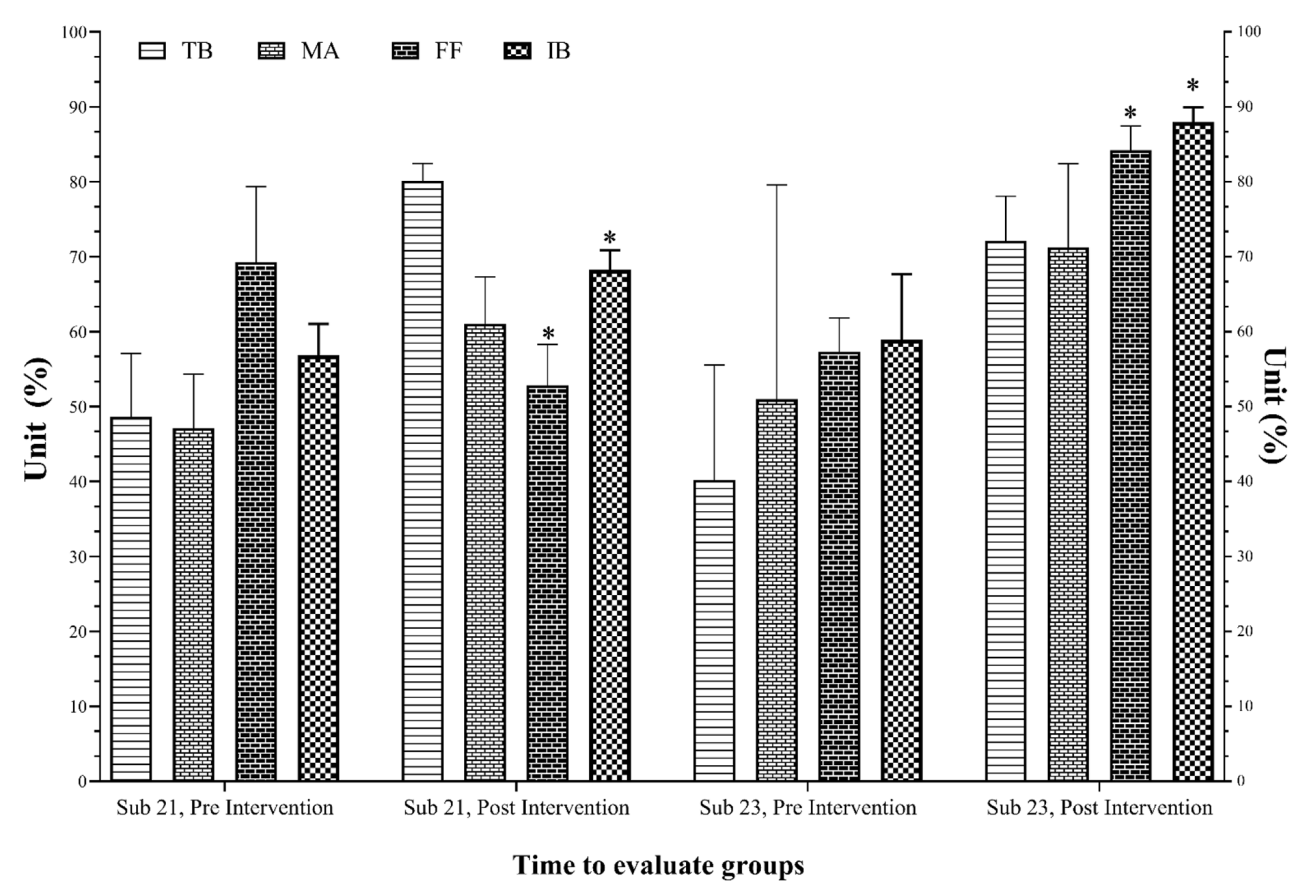
Change in the tactical tests compared based on each category and evaluation stage. TB: Take the ball out of the pressure zone; MA: Mark in attack; FA: First option to pass forward; IB: Immediate pressure when the ball is lost. Indicates a significant change versus the pre-test at a significant level of *p* < 0.05

Regarding game number 3 (i.e., first option to pass forward), it was no significant in main effect of time (*p* = 0.101, F = 3.45, ηp^2^ = 0.301). Whilst it showed significant in the group-by-time interactions for this variable (*p* ≤ 0.001, F = 58.96, ηp^2^ = 0.881). Post hoc analysis demonstrated that game number 3 was significantly greater after intervention than pre-test in both groups (U21: *p* = 0.039 vs. U23: *p* ≤ 0.001). Finally, we found significant changes in the game principle number 4 (immediate pressure when losing the ball) through the main effect of time (*p* ≤ 0.001, F = 105.41, ηp^2^ = 0.929) and group by time interactions (*p* = 0.002, F = 20.08, ηp^2^ = 0.72). Post hoc analysis demonstrated that game number 4 was significantly greater after intervention versus pre-test in both groups (U21: *p* = 0.006 vs. U23: *p* = 0.001). These differences are available in Fig. 2.

The results of the Paired Samples t-test of the combination of both groups showed a significant difference between tactics number one (*p* ≤ 0.001, g = 3.06) and four (*p* ≤ 0.001, g = 2.18) before and after the training intervention (Table 4).

**Table 4:** Tactical components comparison of the participants of both teams in the form of a sample

Variables	*Pre Intervention*	*Post Intervention*	*P*	95% CI of the Difference		Hedge’s g [95% CI values]
				Lower	Upper	
TB (%)	44.39 ± 12.53	76.11 ± 6.03	≤ 0.001*	22.48	40.96	3.06 [1.77 to 4.35]
MA (%)	49.05 ± 19.77	66.13 ± 10.10	0.056	2.33	31.84	1.03 [0.10 to 1.97]
FA (%)	63.29 ± 9.70	68.53 ± 17.06	0.513	-7.81	18.28	0.36 [-0.53 to 1.24]
IB (%)	57.87 ± 6.58	78.12 ± 10.61	≤ 0.001*	11.95	28.55	2.18 [1.07 to 3.28]

CI: Confidence Interval; TB: Take the ball out of the pressure zone; MA: Mark in attack; FA: First option to pass forward; IB: Immediate pressure when the ball is lost. Indicates a significant change versus the pre-test at a significant level of *p* < 0.05

## Discussion

The study’s objective was to test the effects of a pilot intervention program to improve tactical behavior and its potential relationship with psychological variables, including emotional and cognitive components commonly associated with stress, anxiety, and attention span between two elite soccer teams from Argentina. The main results show significant differences, demonstrating the benefits of the intervention program based on implementing strategies related to managing stress scenarios, coping with adverse situations, and developing external attention. The results showed the benefits of the intervention program based on implementing strategies related to managing stress scenarios, coping with adverse situations, and developing external attention. Specifically, both teams showed a significant improvement during the post-intervention compared to the pre-intervention in terms of tactical behavior in competition, except for one of the evaluated game principles (e.g., first-choice forward pass). Therefore, the hypothesis of the present study on the effectiveness of the intervention program is confirmed. Building on the previous point, while we did not directly measure resilient characteristics in this study, previous research has indicated the significance of exposing players to stressful stimuli during specific training tasks, which can enhance their resilient qualities [23]. In our case, the introduction of stressful situations linked to the tactical component may have contributed to the development of resilient characteristics within the teams during both training and competition [24, 25]. As demonstrated in this study, these experiences could have played a crucial role in improving the tactical behavior and game principles of soccer teams during competitions.

In this study, as stress scenarios, coping with adverse situations and foci of attention were introduced in the technical-tactical training tasks, players had to join efforts to solve the relevant tasks and meet the integrated tactical and psychological objectives. In this sense, the increase in the percentage of actions performed in competitions after the intervention would highlight the importance of using strategies with these characteristics. Consequently, it is observed that game principle number 1 (i.e., getting the ball out of the pressure zone) gave the expected results for both teams due to the increase in the percentage of actions performed during the competitions. Specifically, after promoting stress and pressure situations in training, where players, after recovering the ball, had to focus their attention on the farthest spaces of the game and solve immediately, this would indicate that specific attention actions in soccer training tasks under stress and pressure situations, would favor the increase of actions and the improvement of this type of tactical behaviors. Therefore, it can be affirmed that the intervention program produced benefits by increasing the percentage of actions and tactical behaviors associated with taking the ball out of the pressure zone after recovering it.

About game principle number 2 (i.e., offensive marking), the percentage of play actions performed increased significantly after the intervention program. However, unlike game principle number 1, it presented less increase in the percentage of tactical actions performed during the post-intervention period; these results could indicate the need to differentiate the intervention periods on tactical and psychological aspects associated with this game principle. Similar situations have been seen in the lack of time to develop or improve psychological or mental abilities associated with performance through tasks associated with tactical objectives [23]. Consequently, it can be mentioned that the intervention program produced benefits by increasing the percentage of integrated tactical and psychological actions and behaviors associated with attacking marking. Similarly, it would highlight the need to modify the intervention periods or strategies associated with these tactical behaviors during team soccer-specific training tasks to seek more significant benefits and improvements. Therefore, the stimulation of behaviors in which the objective is not only to avoid shots or own goals, together with sacrificial characteristics such as aggressiveness in duels with the attacker and resilience behaviors of the team in adverse situations or pressure and stress scenarios such as staying with fewer players during the task, would favor this principle of play.

In the analysis results in game principle number 3 (i.e., first forward pass option), it is observed that the limitations and strategies used did not cause significant differences, maintaining the percentage of tactical actions performed in the competition during the post-intervention period. These results could indicate that the strategies used are inadequate to improve the tactical and psychological components associated with this game principle. Alternatively, the timing of the intervention program should be modified to achieve more benefits based on game principle number two [26]. At the same time, establishing on-field progression criteria toward the opposing goal could be revised to improve this type of on-field progression principle. To be precise, by analyzing the response to pressure and stress situations in training tasks, such as the maximum number of passes to get from one zone to another in the direction of the “opposing goal”. The specific attention on the sectors and teammates to deliver the ball, such as adding a goal for each pass made towards the next playing zone). It would be necessary to deepen the strategies and timing used to improve this principle of play.

When analyzing game principle number 4 (i.e., immediate pressure upon loss of the ball), it is observed that the strategies and conditioning used favoured an increase in the percentage of tactical and psychological actions during competitions after the intervention. Similar behavior is observed for game principle number two, where an increase in the percentage of game actions performed during the post-intervention period is observed. Consequently, the established hypothesis can be confirmed since a higher percentage of tactical actions performed in competitions is observed after the intervention program. Specifically, this could be corroborated during the post-intervention periods compared to the pre-intervention period.

Concerning the strengths of the present study, a quasi-experimental design was used with two teams of different ages. In this way, it was possible to recognize the importance of the timing of the intervention program in two teams with a difference in terms of ages of soccer experience. In this sense, it could be fundamental to differentiate the moment of intervention on the technical-tactical aspects of the game from the psychological aspects, according to the level of competition or stage of training/performance of the soccer players. Even so, these results reaffirm what other studies have posited regarding intervention programs, where those with a duration of between 2 to20 weeks showed the most remarkable significant changes [27]. Therefore, establishing interventions with a duration aligned with the adaptation and improvement processes could achieve the desired results. Fundamentally, as a product of an intervention developed during training and specific game tasks, where it could be reaffirmed as one of the most effective strategies [28]. In turn, this study proposes interventions in team training associated with improving tactical and psychological components integrated through concrete strategies in technical-tactical training tasks [29].

Beyond the study’s strengths, it is necessary to point out some limitations. Initially, few participants were included, and a control group was not established. It would have allowed corroboration of the differences between the control and experimental group. In the future, it would be interesting to conduct research with a mixed analysis of quantitative and qualitative data to examine the program’s effects [30] on measures of play and external instruments used with questionnaires and psychological inventories. Along these lines, intervention programs with a duration like the planning of an entire season could be recommended. In this way, it would be possible to know the behavior over more extended periods, corroborating whether the benefits are maintained over time or whether it is necessary to maintain the strategies as occurs with the rest of the planned components (e.g., physical, and technical).

The absence of test-retest reliability assessments for the outcome measures indeed presents a limitation in the study’s methodology. It’s imperative to acknowledge that this absence raises concerns regarding the consistency and stability of the measurements taken over time. However, it’s noteworthy that each group in the study had a control period of five weeks before the five-week intervention. This design, incorporating repeated measures within each group, does to some extent address the absence of a typical control group. While acknowledging the limitation of not conducting test-retest reliability assessments, it’s crucial to recognize that the study’s design attempted to mitigate this limitation by implementing a controlled pre-intervention period for each group. This setup allows for within-group comparisons, offering insights into the changes occurring within each group over time. Therefore, although test-retest reliability wasn’t explicitly assessed, the design does provide a comparative framework within the study’s context.

It’s essential to interpret these findings considering the absence of formal test-retest reliability assessments. The control period before the intervention offers a baseline for comparison within each group, contributing to understanding the effects of the intervention. However, the absence of direct test-retest measurements may limit the extent to which we can confidently attribute observed changes solely to the intervention, warranting cautious interpretation of the study’s outcomes.

## Conclusions

Based on the study results, we conclude that the intervention program on tactical/psychological aspects integrated and focused on improving the principles of play of two soccer teams through technical-tactical training tasks showed benefits in the teams’ performance during competitions. These results extend previous knowledge where the importance of working under stressful situations and coping with adverse situations in training was highlighted. In this sense and regarding the practical applications extracted from this study, coaches, psychologists, and physical trainers can use the strategies proposed in this research to improve tactical behavior in competition. In addition, this intervention program has shown that tactical and psychological components can be planned within the usual technical-tactical training tasks in an integrated and specific manner for game situations.

## Data Availability

The datasets generated during and analyzed during the current study are available from the corresponding author.
